# Supplementary material for: A Global Screen for Assembly State Changes of the Mitotic Proteome by SEC-SWATH-MS
Source: Cell Syst. 2020 Feb 26;10(2):133–155.e6. doi: 10.1016/j.cels.2020.01.001 (PMC7042714; doi:10.1016/j.cels.2020.01.001)

Q9Y2K7 | KDM2A_HUMAN | KDM2A CXXC8 FBL7 FBXL11 JHDM1A KIAA1004
Monomer MW [kDa]: 132.793 Monomer expected elution fraction: 38

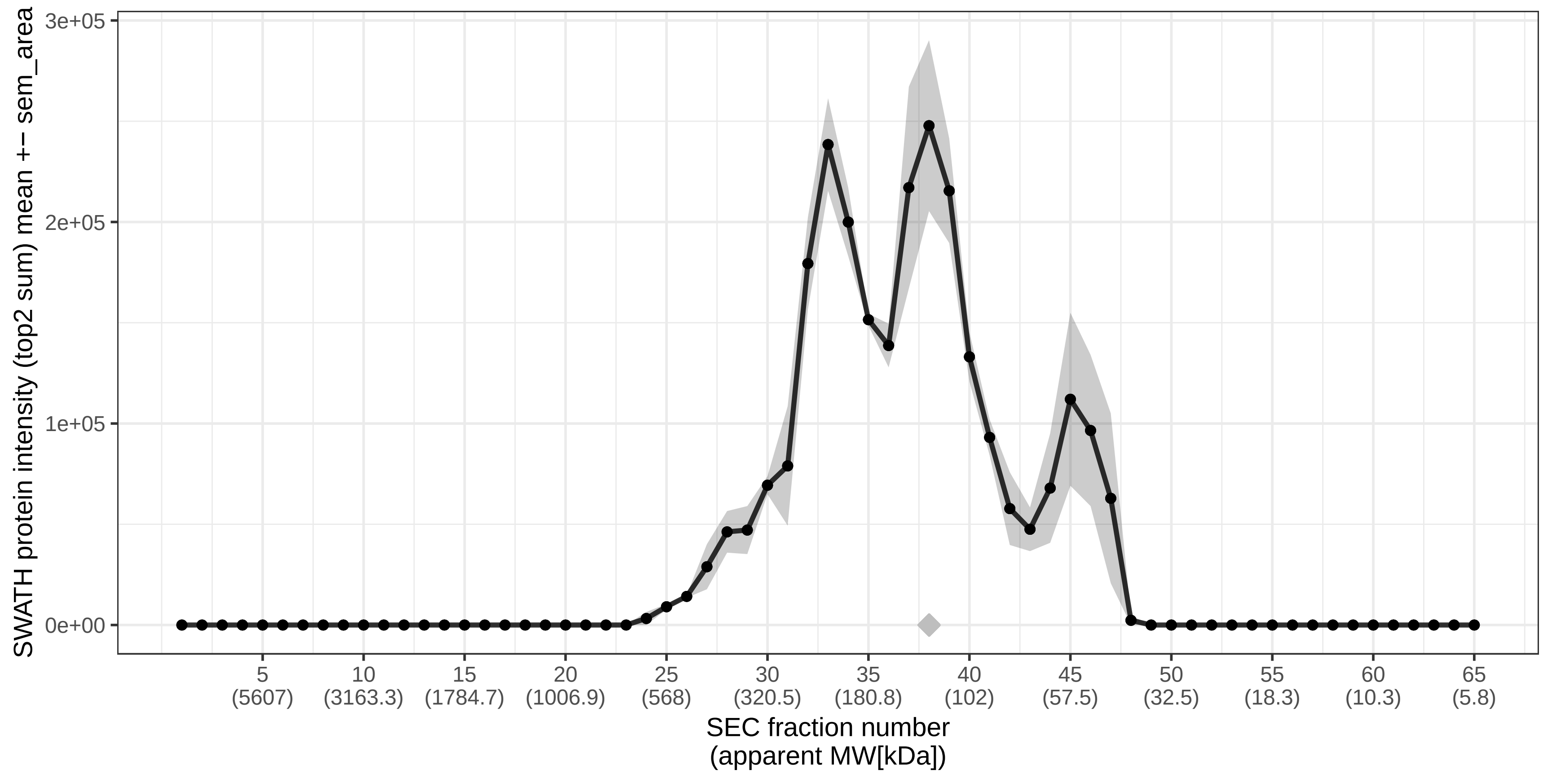

Supplement: Data S1. SEC-SWATH-MS Protein Chromatograms, Related to Figure 1 [file mmc6.zip › SECchrom_Q9Y2K7_KDM2A_HUMAN_KDM2A_CXXC8_FBL7_FBXL11_JHDM1A_.pdf]
